# Manifold-based sparse representation for opinion mining

**DOI:** 10.1038/s41598-023-43088-9

**Published:** 2023-09-23

**Authors:** Zohre Karimi

**Affiliations:** School of Engineering, Damghan University, Damghan, Iran

**Keywords:** Engineering, Computer science

## Abstract

What the consumer thinks about an organization's products, services, and events is a crucial performance indicator for businesses. The brief opinion pieces were quickly published on websites and social media platforms and have been analyzed by machine learning methods. The classical text feature representation methods suffer from high dimensionality, sparsity, noisy, irrelevant and redundant information. This paper focuses on how to enhance feature representation for opinion mining. Some nonlinear feature selection methods based on manifold assumption have been exploited to resolve these problems. The inherent manifold configuration was commonly ascertained through a nearest neighbor graph, whereby the neighbors in the current techniques may exhibit diverse polarities. To alleviate this burden, it is proposed to exploit both manifold assumption and sparse property as prior knowledge for opinion representation to learn intrinsic structure from data. First, the graph representation of user reviews based on the mentioned prior knowledge is learned. Then, the spectral properties of the learned graph are exploited to present data in a new feature space. The proposed algorithm is applied to four various common input features on two benchmark datasets, the Internet Movie Database (IMDB) and the Amazon review dataset. Our experiments reveal that the proposed algorithm yields considerable enhancements in terms of F-measure, accuracy, and other standard performance measures compared to the combination of state-of-the-art features with various classifiers. The highest classification accuracies of 99.15 and 91.97 are obtained in the proposed method on IMDB and Amazon using a linear SVM classifier, respectively. The impact of the parameters of the proposed algorithm is also investigated in this paper. The incorporation of a sparse manifold-based representation has led to noteworthy advancements beyond the baseline, and this success serves to validate the underlying assumptions.

## Introduction

The availability of user-generated information from online or offline documents has achieved considerable growth in the past few years since the emergence of online social network platforms, web communities, blogs, wikis, and other social media^1,2^. This growth indicates the increasing need for analyzing user-generated opinions. Opinion mining, also known as sentiment analysis, is one of the emerging applications of textual data analytics, natural language processing and computational linguistics to specify the orientation of a particular opinion, usually as positive, negative, or neutral polarities.

The short opinion text generated by users has become an essential source of information about people's thoughts and feelings on anything or any events. As a result, it plays a crucial role in a wide range of applications in many genres, from marketing to medicine^3–7^. The polarity detection methods in opinion mining literature can be divided into three groups of document, sentence, and aspect level based on their granularity; these methods predict the attitude of each review, each sentence, and each aspect, respectively. From another perspective, opinion mining can be divided into computational linguistic and machine learning methods. The first approach utilizes the linguistic background, and the polarity of the review is specified by computing its score based on the occurrence of lexical words. It assumes that positive words appear with a higher probability than negative words in a positive sentiment. A modern approach applies machine learning methods by learning classifiers. Popular classifiers, including support vector machine (SVM), Naïve Bayes, neural network^8,9^, Bayesian network and Adaptive Neuro-Fuzzy Inference System^10^ which are successful classifiers in various applications are used to specify the attitude of the opinions^11,12^. In this approach, sentiment analysis can be viewed as a binary classification problem. It shows better prediction performance than the former methods because labeled data sampled by domain experts are used to train a model. However, the performance of the trained classifier is significantly dependent on the features produced from unstructured texts^13^.

**Figure 1:**
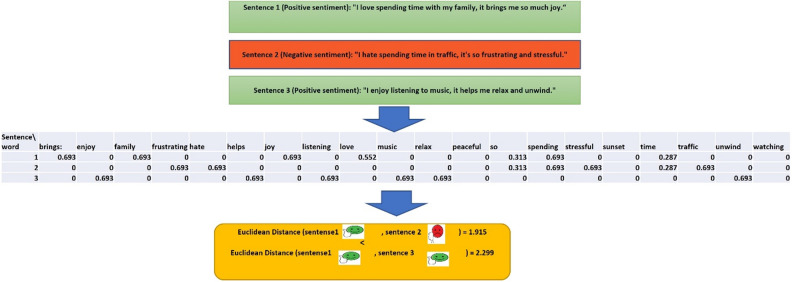
The Euclidean distance of TF_IDF vectors of sentence 1 and 2 with dissimilar polarities (after removing stop words) is less than the Euclidean distance of TF_IDF vectors of sentence 1 and 3 with similar polarities.

Well-known features such as unigrams, bigrams, etc. embed text into a vector space. The vectors representing text are high-dimensional feature vectors consisting of noisy, redundant, and irrelevant features and suffer from data sparsity. Therefore, feature representation is of prime importance in machine learning methods. The feature selection and feature extraction are two main approaches to handle the mentioned problems. Feature selection methods select a subset of relevant features, whereas feature extraction methods transform a large number of features into a new set of features in a different space. Feature extraction methods have higher discriminating power and less information loss than feature selection methods^14^. However, there are some problems in the current application of feature extraction methods with opinion mining. Some methods are linear which can’t reflect the non-linear properties of high dimensional sentiment data. There is plenty of research confirming that high dimensional data lie close to the manifold structures^15–19^. Recently, classical linear methods for feature extraction and reduction have been generalized to the nonlinear manifold techniques by establishing a correspondence between a high dimensional space and an intrinsic structure by considering topological relationships. A vast amount of machine learning literature has focused on this line of research^17–22^, whereas only a little attention has been dedicated to this field in opinion mining literature. The intrinsic structure of data is represented by graph structure and making suitable graph is highly effective in the result of manifold-based methods. In the literature of opinion mining, the graph structure usually is constructed by $k$-nearest neighborhood or $\in$-nearest neighborhood graph, where the nearest neighborhood of each data is specified by common distance measures such as Euclidean or cosine distance^23,24^. Furthermore, some linguistic features like co-occurance is also exploited for building graph in the manifold-based sentiment analysis^25^. It is prime of importance that graph represents the manifold structure of opinion data and the existing either some wrong strong connections between dissimilar local data or some weak connections between similar local data lead to the error in the next algorithm’s steps. The common manifold-based methods suffer from the mentioned error. For more clarity, the drawback of using Euclidean distance for specifying locality is illustrated in Fig. 1. Sentences 1 and 2 exhibit positive polarities, while sentence 3 demonstrates a negative polarity. However, it is noteworthy that the Euclidean distance between sentence 1 and 3, which possess similar polarities, is lesser than the distance between sentence 1 and 2, which have dissimilar polarities. Other common feature representation methods and distance measures, which mentioned earlier, also have not addressed this issue sufficiently.

This paper focuses on document-level sentiment analysis and attempts to resolve the above challenge by assuming that input sentiment vectors lie on some manifolds. The motivation of this assumption is the line of research that demonstrate high dimensional data lies on some interesting manifolds against to only one manifold^16,19^. It means that some near opinions, based on common distance measures, may have various attitudes, and it is needed to another prior knowledge is imposed to resolve the problem. Therefore, an algorithm based on sparse representation technique, i.e., Sparse Manifold Sentiment Representation (SMSR), is developed and studied to represent sentiment data based on the two following properties of high dimensional data: (1) self-expressiveness property of the data, which reflects that each data point in a union of subspaces can be expressed as a linear combination of other data points^26^, (2) sparse representation of a data point matches to a combination of a few data points locating in its own subspace^20^.

Sparse representation has led to promising results in a wide range of applications, including visual recognition^27^, image synthesis^28^, animation^29^, denoising^30^, etc. Recently, sparse representation is applied to sentiment analysis^31,32^. ${l}_{\mathrm{2,1}}$-norm sparsity is exploited to represent micro-videos with the aim of finding the main frame including sentiment^33^. The singular vector decomposition is also used for the sparse representation of image sentiments^31^. The success of sparse learning in sentiment analysis on image and video and the importance of graph representation in manifold-based feature extraction are the author’s motivation for incorporating them together.

The proposed algorithm learns the graph representing manifolds from data by using both sparse and locality representation and then exploits spectral properties to generate a new data representation.

The main contributions of this paper are described as follows:

1. The feature extraction on sentiment data is applied by considering laying sentiment data on some manifolds.
2. The manifolds’ structure of opinions is discovered by both local information and sparse properties of data.
3. The impact of sparse and manifold-based representation on some common feature extraction methods of sentiment analysis is explored to provide insights to various vector representation. Our experiments confirm the enhancement of the proposed method compared to all of them.
4. The effect of the proposed method parameters is investigated, and the obtained results demonstrate the validity of the proposed method in a wide range of values.
5. The linear SVM classifier suitable for text data is applied to the extracted features on two benchmark datasets. To the best of our knowledge, a wide range of research has approved the performance of SVM classifiers compared to other classifiers. This paper exploits SVM with linear kernel that has fewer parameters compared to SVM with both Radial basis function and polynomial kernel. Numerous experiments reveal that extracted features are highly suitable features for this classifier.

It is noteworthy to mention that the last three cases mentioned above highlights the main aspects of this research in comparison to our previous research^34^. This paper combines the key ideas from sparse representation, manifold learning, and the sentiment analysis.

The rest of this paper is organized as follows. “Literature review” section examines the literature review. Then, “proposed method” describe the details of the proposed algorithm. The details of experiments and results are given in the fourth section, followed by a conclusion in the end.

## Literature review

In recent years, opinion mining has become an active research area^35,36^. In this section, some research on enhancing sentiment analysis performance by feature representation methods, including feature selection and feature extraction, has been examined.

Feature selection methods in opinion mining literature falls into three categories of natural language processing (NLP) based, machine learning-based and the combination of them. NLP-based techniques exploit lingual characteristics such as nouns, noun phrases, adjectives, and adverbs as features^37,38^. NLP-based techniques have achieved high accuracy but low recall, depending on the amount of accuracy of part of tagging speech.

The feature selection methods based on machine learning are divided in three categories including filter approaches, wrapper approaches, and hybrid methods. In filter approaches, a score is assigned to each feature based on the evaluation function and the features with low scores are eliminated. This approach has low computational cost and easy to implement. Feature filtering methods such as information gain (IG), chi-square (Chi2), occurrence frequency, Z-score, logarithm likelihood, fisher discriminant ratio and minimum frequency thresholds consider attributes separately^39–45^. The document frequency (DF) feature selection method is commonly used in the general text classification and picks the most common terms in the corpus. Mutual Information, IG, Chi2, and DF with five machine learning algorithms, consisting of support vector machine, $k$-nearest neighbor, centroid classifier, naïve Bayes, and winnow classifier, were applied for sentiment analysis of Chinese documents^39^. Cekik et al. proposed a feature selection method using rough set theory to handle sparse opinion data^46^. Wang et al. exploited an improved fisher discriminant ratio for feature selection of sentiments^41^. Filter-based techniques have computational efficiency, but they neglect the interactions between features^47^.

In wrapper methods, a feature is either added or removed at each step to select an optimal feature subset in a greedy manner. When a new feature set is selected, the classifier is retrained with new features and assessed on a validation set. These methods suffer from high computational cost. Some important wrapper methods are decision tree models, recursive feature elimination, and heuristic based algorithms^48^. The wrapper methods are more expensive than filter models in terms of computational efficiency, since they evaluate attribute interactions. Gokalp et al. proposed a wrapper feature selection method using iterated greedy metaheuristic algorithm for opinion mining^49^. Hybrid techniques combine other groups for exploiting their suitable properties and achieving both accuracy and efficiency^43,50,51^. Hybrid or embedded methods find a subset of features in the machine learning methods.

Feature extraction methods can be grouped into linear or nonlinear methods. Linear methods are based on the simple assumption of linearity. Latent semantic analysis and principal component analysis are two popular methods applied to opinion mining^52,53^. The implementation of linear dimensionality reduction is easy and fast. Meanwhile, they cannot represent complex nonlinear data appropriately. Few studies on opinion mining consider the nonlinear structure of data which are local methods applying manifold assumption by enforcing near data points in the input space, close to each other in the new feature space. Kim et al. exploited semi-supervised Laplacian eigenmaps to reduce the dimensionality of data and visualize sentiments^24^. This method is based on manifold regularization utilizing unlabeled data in the training step. Ma et al. proposed another semi-supervised nonlinear feature extraction based on Laplacian eigenmap, where its initial graph is obtained using fuzzy similarity relation^54^. A semi-supervised dimensionality reduction combined with feature weighting is also proposed in^25^. It attempts to maximize between-class distance and minimize within-class distance, exploiting the local structure of unlabeled data at the same time.

The summarization of the literature is given in Table 1. The focus of this paper is on the representation of opinion data using a nonlinear feature extraction method by considering laying data on some manifolds and discovering this structure by sparse properties, which have neglicated in the literature. The significance of constructing graphs in manifold-based methods has been studied and demonstrated in a number of research studies. Some methods exploit label information to make a discriminant graph with the aim of classification. Discriminant neighborhood embedding (DNE) method has been introduced an optimization problem for learning a discriminant embedding by considering both intra-class and inter-class adjacency weights, where these adjacency weights are simply in $\{\mathrm{0,1}\}$ ^55^. Some other researches improved DNE by extending both weight values and neighborhood definition^21,56^. A theoretical framework for improving the graph weights’ learning has also been proposed in^21,22^. The mentioned methods have been evaluated on image datasets.

**Table 1:** The summarization of related dimensionality reduction methods in sentiment analysis.

Dimensionality reduction method	Ref	Techniques	Advantage	Disadvantage
Feature selection	^41^	Exploiting fisher’s discriminant ratio	Easy to implementing, fast	Neglect the interactions between features
	^43^	Filter using mutual information or point-wise mutual information		
	^39^	Mutual Information, information gain, chi-square, and document frequency		
	^41^	improved fisher discriminant ratio		
	^76^	Information gain		
	^43^	Wrapper-based by genetic algorithm	Take into account the relations between features	Neglect the manifold structure of high dimensional data
	^49^	iterated greedy metaheuristic algorithm		
Feature extraction	^53,76^	PCA	Fast, easy to implement	Consider linearity assumption
	^52^	PCA, LDA		
	^24^	Semi-supervised Laplacian eigenmap	exploit local label information and consider non-linearity of data	Consider lying data on one manifold
	^25^	Exploiting Laplacian of graph made from data in the combination with feature weights	consider locality of data by assigning weights to features	consider certain types of prior knowledge or directly utilize label information to construct a weighted graph
	^23^	ISOMAP ^77^	Exploit semantic and sentiment distance between opinions by computing cosine distance between the opinion’s vectors and the difference in the degree of sentiments, respectively	Consider lying data on one manifold

Similar to existing nonlinear methods^24^, a graph is constructed from data, but instead of using $k$-nearest neighbor ($k$ NN) or $\epsilon$-nearest neighbor with some heuristics^24,54^, a graph is learned from data in this paper in order to detect close data points by imposing sparsity constraint. Sparse representation successfully applied to various applications. Recently, it has been an active research area in the sentiment analysis. ${l}_{\mathrm{2,1}}$-norm sparsity in the incorporation of the regularization term based on Laplacian matrix is exploited to extract sentiment key frame extraction in user-generated micro-videos^33^. The cosine distance between video frame vectors is used to compute the graph weights. The sparse representation by singular vector decomposition is used to enhance the efficiency of deep learning in image sentiment analysis. It ignores the local smoothness assumption as an effective prior knowledge^31^.

In the comparison of the above sparse research on sentiment analysis, the focus of the proposed method is on short text data and attempts to learn a suitable data dependent graph structure.

## The proposed method

### Problem formulation

**Figure 2:**
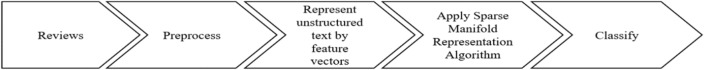
The steps of the proposed method.

Let $\mathcal{R}={\left\{{r}_{i}\right\}}_{i=1}^{N}$ be a collection of N reviews, where ${r}_{i}$ is $i$ th opinion. A sentiment label is assigned to each review of ${r}_{i}$, in the labeled training set and is shown by ${y}_{i}$ that can be + 1 (positive) or -1 (negative), 0 is also may be included indicating neutral sentiment but it is not considered in this research. Table 2 gives the main notations of this paper. The ultimate aim of opinion mining is to predict the sentiment label of reviews with unknown attitudes. The essential steps of the proposed method are given in Fig. 2.

**Table 2:** Notations.

Symbol	Description
D	Dimension of input data
N	The number of input reviews
d	The dimensionality of data in the learned feature space
$\mathcal{X}={\left\{{x}_{i}\in {\mathbb{R}}^{D}\right\}}_{i=1}^{N}$	a set of N feature vectors, where vector ${x}_{i}$ represents features of $i$ th review
${y}_{i}\in \{+1,-1\}$	The attitude of ${x}_{i}$
$\lambda$	The coefficient of sparsity term in the optimization problem
$k$	The maximum neighborhood size to select the sparse neighbors
${\varvec{L}}$	Laplacian Matrix
${\varvec{W}}={\left[{w}_{i}\right]}_{i=1}^{N}$	Weight Matrix
${\varvec{D}}$	Degree Matrix
${{\varvec{X}}}_{{\varvec{i}}}\in {\mathbb{R}}^{D\times N-1}$	The matrix of normalized vectors
${\mathcal{M}}_{l}$	$l$ th manifold

### Research motivation

Let $\mathcal{X}={\left\{{x}_{i}\in {\mathbb{R}}^{D}\right\}}_{i=1}^{N}$ be a set of review vectors, ${x}_{i}$ is the feature vector of representing $i$ th review, ${r}_{i}$, and can be extracted by various methods, including statistical, lexicon-based, and combined. The details of feature extraction methods in this paper are given in “Extract feature vectors from unstructured text” subsection** .** The features generated from unstructured reviews consist of redundant, sparse, and unusual information and should be enhanced for the opinion mining task. Some researches are exploited manifold assumption and reduce the dimensionality of opinion data by constructing a locality graph from data. Nonetheless, By studying various methods of nonlinear dimensionality reduction, three following achievements are available:

1. High dimensional data lies on some intersecting manifolds.
2. The accurate graph construction of underlying manifolds is so important, especially in the intersecting regions.
3. Sparse representation can find the representation of one data based on other data points in its subspace (local tangent space)

Therefore, it is assumed that some nonlinear manifolds of opinions are embedded into the high dimensional input space, and a few nearest neighbors can construct each data point in its manifold. The weights of this reconstruction for all data are learned by imposing the mentioned assumptions as prior knowledge. It is proposed to exploit sparse manifold based representation to efficiently and accurately analyze high-dimensional data by restoring its low-dimensional structure. After learning graph structure from data, spectral methods are exploited to extract the new representation. The details are explained in “Sparse Manifold Representation Algorithm” subsection.

**Figure 3:**
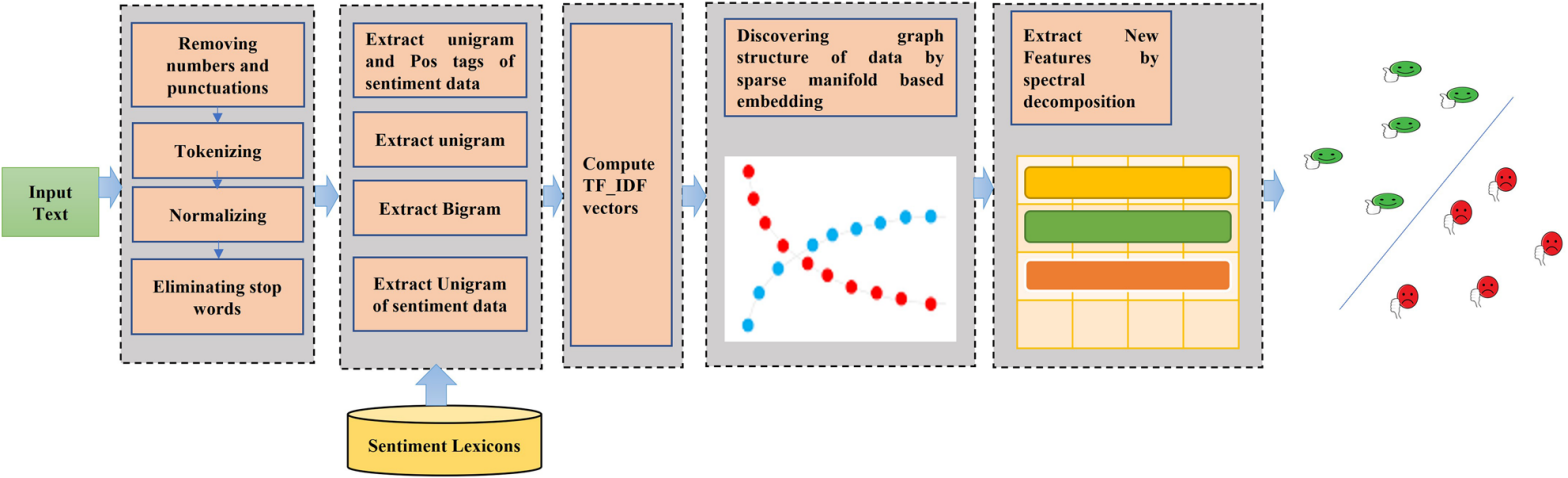
The architecture of proposed method.

Finally, a classifier is applied to learn a model that discriminates positive attitudes from negative ones in the feature space and predicts the overall attitude within unknown labels. The architecture of the proposed method is shown in Fig. 3.

### Extract feature vectors from unstructured text

Some preprocessing steps are carried out at the first stage, including removing numbers and punctuations, normalizing, tokenizing, and eliminating stop words. Then, the commonly used features are extracted in the phase of extracting features. The popular features in the literature are n-gram, parts of speech (POS) tagging, term frequency-inverse document frequency (tf-idf), and their combination^39,57,58^. The mentioned features are used in our experiments as input features and are described as follows:

- n-gram: An n-gram is defined as a subsequence of n words from a given sequence by eliminating extra spaces and noisy characters between two words. Unigram or bag of word model is the simplest model and consists of all the individual words presented in the text. The bigram model is defined as a pair of adjacent words and can include some contextual information. Higher orders of n-grams are more efficient in capturing the context as they provide a better understanding of the word position, while their generation is inefficient. The unigram (n = 1), bigram (n = 2) and trigram (n = 3) are commonly used n-gram models.
- POS tagging: POS tagging is a linguistic technique that gives a tag to each word by specifying its morphological category such as noun, verb, adjective, etc.
- Lexicon-based features: A list including positive and negative words with their scores is provided in a sentiment lexicon. An n-gram is considered in the features if it is provided in the list with acceptable scores.

The terms are specified based on one or more features above and weighted according to the tf-idf weighting method. Intuitively, tf-idf specifies how relevant a given term is in a particular document. It consists of two parts: the frequency of terms in a specific review, and the inverse proportion of that term over the entire review. Equation (1) is the classical formula of tf-idf related to $i$ th term in $j$ th document (review):

1 $${tf-idf}_{i,j}=t{f}_{i,j}\times log\frac{N}{d{f}_{i}}$$

where $t{f}_{i,j}$ indicates the term frequency of term $i$ in $j$ th review and $d{f}_{i}$ is the document frequency of $i$ th term in all reviews. Terms that occur in one or a small set of documents are given more tf-idf weight than terms that occur in many documents. Finally, $i$ th review is represented by ${x}_{i}=({x}_{i1}, \dots ,{x}_{iD})$, where ${x}_{ij}={tf-idf}_{i,j}$.

There exist alternative techniques for encoding opinion texts into vectors. One such approach is Doc2Vec^23^, which employs a neural network to generate a vector representation of each opinion of a fixed length. The learning process is designed to map similar documents to proximate points within the vector space. The resultant vectors can be effectively leveraged within the proposed method.

### Sparse manifold representation algorithm

**Figure 4:**
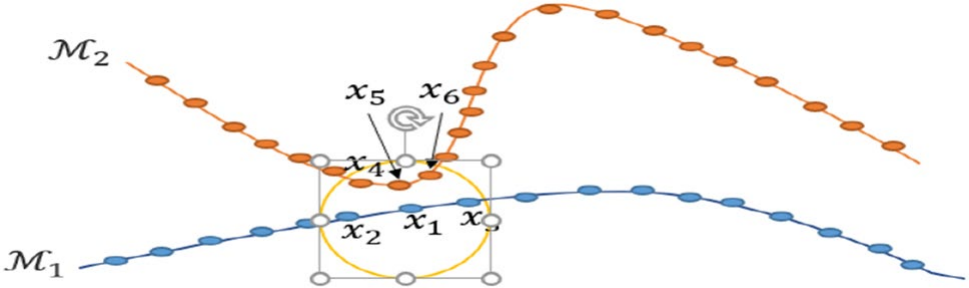
${\mathrm{x}}_{2}$ and ${\mathrm{x}}_{3}$ are far from ${\mathrm{x}}_{1}$ in the comparison of ${\mathrm{x}}_{4}$, ${\mathrm{x}}_{5}$, ${\mathrm{x}}_{6}$ lying on different manifolds, whereas all of them are in the nearest neighbor of ${\mathrm{x}}_{1}$. The sparse optimization selects ${\mathrm{x}}_{2}$ and ${\mathrm{x}}_{3}$ to reconstruct ${\mathrm{x}}_{1}$ which span a 1-dimensional affine subspace passing near ${\mathrm{x}}_{1}$ and approximates its manifold (Adapted from^20^).

After the step of representing reviews by feature vectors, there is a set of N data points $\mathcal{X}=$ ${\left\{{x}_{i}\in {\mathbb{R}}^{D}\right\}}_{i=1}^{N}$ lying in n different manifold ${\left\{{\mathcal{M}}_{l}\right\}}_{l=1}^{n}$ and the aim is to discover a new representation of the data by the sparse manifold embedding method considering these two assumptions^20^: (1) each data point ${x}_{i}\in {\mathcal{M}}_{l}$ can be reconstructed by the linear combination of its neighbors, and (2) the minimum of these neighbors from the same manifold is formed by the sparse representation of ${x}_{i}$. In other words, between the infinitely many possible representations of a data point which use other data points, a sparse representation finds a few points from the same subspace. These assumptions are illustrated in Fig. 4, where points ${x}_{2}$ and ${x}_{3}$ are farther than ${x}_{4}$, ${x}_{5}$ and ${x}_{6}$ to ${x}_{1}$, whereas they lie on the different manifold of ${x}_{1}$, ${\mathcal{M}}_{2}$, and ${x}_{4}$, ${x}_{5}$ and ${x}_{6}$ are located in any small ball centered at ${x}_{1}$ containing ${x}_{2}$ and ${x}_{3}$. Nevertheless, only ${x}_{2}$ and ${x}_{3}$ span a one-dimensional subspace around ${x}_{1}$ which is close to the tangent space of ${\mathcal{M}}_{1}$ at ${x}_{1}$ and can be found by the sparse constraint.

This idea is studied in both machine learning and image classification literature, and to the best of the author's knowledge, so far, less attention has been paid to the sentiment analysis application^20,59,60^. Manifolds are represented by a graph structure so that each data point ${x}_{i}$ is a node of the graph and the weights of connections between $i$ th node and other nodes formed by the vector of ${c}_{i}\in {\mathbb{R}}^{N-1}$. ${c}_{ij}$ is the weight between ${x}_{i}$ and $j$ th element of $\mathcal{X}$ without considering ${x}_{i}$. ${c}_{i}$ are obtained by the reconstruction of each data point based on two mentioned properties of proximity and sparsity by the following optimization problem^20^:

2 $$\begin{aligned}{}& \mathop {\min }\limits_{{{\text{c}}_{{\text{i}}} }} \;\lambda \left\| {Q_{i} c_{i1} } \right\| + \frac{1}{2}\left\| {X_{i} c} \right\|_{2}^{2} \\ & {\text{Subject}}\;{\text{ to }}\;1^{T} c_{i} = 1 \\ \end{aligned}$$

where l1-norm, ${\Vert .\Vert }_{1}$, imposes the sparsity constraint, $\lambda >0$ is a given parameter used for tradeoff between the sparsity against the reconstruction error. ${Q}_{i}$ is a positive-definite diagonal matrix whose smaller values show the nearby points to ${x}_{i}$ and larger values indicate farther points from ${x}_{i}$, preferring the zero values for them. A common definition of ${Q}_{i}$ is as:

3 $${Q}_{i}\triangleq diag\left({\left[\frac{{\left|\left|{x}_{j}-{x}_{i}\right|\right|}_{2}}{\sum_{t\ne i}{\Vert {x}_{t}-{x}_{i}\Vert }_{2}}\right]}_{j\ne i}\right)\in {\mathbb{R}}^{N-1\times N-1}$$

Furthermore, other alternatives for ${Q}_{i}$, exponential weights for example, maybe exploited; ${X}_{i}$ is the matrix of normalized vectors ${\left\{{x}_{i}-{x}_{j}\right\}}_{j\ne i}$ as

4 $${X}_{i}\triangleq [\frac{{x}_{1}-{x}_{i}}{{\Vert {x}_{1}-{x}_{i}\Vert }_{2}}\dots \frac{{x}_{N}-{x}_{i}}{{\Vert {x}_{N}-{x}_{i}\Vert }_{2}}]\in {\mathbb{R}}^{D\times N-1}$$

The optimization problem (2) is a Lasso optimization problem with the additional affine constraint^61^.

The coefficient vectors of ${\left\{{c}_{i}\right\}}_{i=1}^{N}$, obtained in the optimization problem (2) are translated into graph edge weight matrix, ${\varvec{W}}=[{w}_{ij}]$, capturing the manifold structure of data. Let ${c}_{i}=[{c}_{i1}\dots {c}_{iN-1}]$, the ${w}_{ij}$ is obtained via the following relation:

5 $$w_{ij} = \left\{ {\begin{array}{*{20}l} {c_{ij} } \hfill & {j < i} \hfill \\ 0 \hfill & {j = i} \hfill \\ {c_{ij - 1} } \hfill & {j > i} \hfill \\ \end{array} } \right.$$

that places the learned weights for the $i$ th data, ${c}_{i}$, in its suitable index of weight matrix. To describe precisely, each node i, representing $i$ th data point, is connected to all other nodes by utilizing the elements of a sparse solution ${c}_{i}$. It is expected that the nonzero elements of ${c}_{i}$ originate from the same manifold. Consequently, the resulting graph should ideally exhibit multiple connected components. This is due to the fact that points within the same manifold are connected to each other, while no connections exist between points in different manifolds (In reality, there exists some weak connections between various manifolds). The obtained graph edge weights are established to connect each data point ${x}_{i}$ to the specified neighbors. In practice, for more efficiency, the learning of weights of each ${x}_{i}$ is restricted to its nearest neighbors, and a parameter $k$ specifies maximum neighborhood size to select the sparse neighbors. Ultimately, a symmetrizing step is conducted to make the obtained weight matrix symmetric. Then, the spectral properties of the obtained weighted graph are utilized to learn a novel representation of data.

The eigenvectors of an affinity matrix are utilized to reveal the intrinsic data structure by three standard steps: (1) Compute the normalized symmetric graph Laplacian^62^

6 $${\varvec{L}} = {\varvec{I}}-{{\varvec{D}}}^{\left(-\frac{1}{2}\right)}{\varvec{W}}{{\varvec{D}}}^{\left(-\frac{1}{2}\right)}$$

where ${\varvec{W}} = [{w}_{ij}]$ is the weight matrix, and ${\varvec{D}}$ is degree matrix as diagonal matrix as

7 $${\varvec{D}}=Diag\left({d}_{1},\dots {d}_{N}\right)$$

8 $${d}_{i}=\sum_{j=1}^{N}{w}_{ij}$$

Other versions of Laplacian can also be used. (2) Compute the d eigenvectors ${v}_{1} . . . {v}_{d}$ of ${\varvec{L}}$ with the smallest eigenvalues. (3) Form matrix ${\varvec{V}}$ with ${v}_{1} . . . {v}_{d}$ as columns. $i$ th row of **V** is the projection of the ${x}_{i}$ onto smallest eigenvectors and are used as the new representation of ${x}_{i}$. The steps of SMSR are given in Alg. 1.

The ultimate goal of sentiment analysis is to predict the attitude of sentiments. Conventional classifiers could be applied to the new representation of data. However, as it will be shown in the next section, if a few eigen vectors are chosen, a wide range of suitable information is lost; therefore, suitable results are achieved by exploiting a classifier that supports high-dimensional data. SVM is a successful classifier that achieved proper results in the opinion mining literature^13,63–65^. In this study, the linear SVM is applied that is appropriate for text application and has fewer parameters than SVM with other kernels^66,67^.
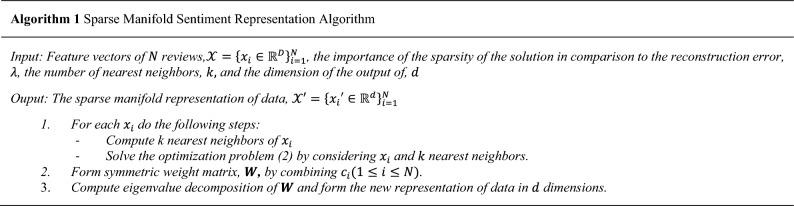


## Experimental analysis and results

### Datasets

The proposed method is trained and tested on two famous datasets: (1) Internet Movie Database (IMDB), including 50,000 movie reviews consisting of 25,000 positively annotated and 25,000 negative labeled reviews (It is available at http://www.cs.cornell.edu/people/pabo/movie-review-data/). ^68^ (2) Amazon review dataset released in 2014 and updated in 2018 and contained 233.1 million reviews and their metadata (The subset of original dataset is downloaded from https://drive.google.com/file/d/1EW-2ZiC2Df8PufsuNMPqIrdx6_zo29D1/view?usp=sharing (last access data: 9/21/2021)) . Some subsets of datasets are randomly selected, including equal positive and negative classes with various features settings. Then, the SMSR algorithm is applied to widely used features including unigrams, bigrams, tagged terms, and sentiment terms as the following four combinations: (1)Unigram + lexicon + tag: the combination of unigrams and POS tags is considered as features, and their tf-idf are computed. (2)Unigram: The tf-idf of unigrams is computed. (3) Bigram: The tf-idf of bigrams is calculated. (4) Unigram + lexicon: Sentiwordnet^69^, a popular sentiment lexicon including positive and negative terms based on their part-of-speech tags, is used in this setting. Sentiwordnet is frequently exploited in the opinion mining literature^70^. In our experiments, unigrams are filtered using sentiwordnet lexicon. Those words whose difference between the sum of positive and the sum of negative scores is less than a pregiven threshold are eliminated and tf-idf of remaining unigrams is computed. The threshold is set to be 0.3.

In all the above settings, unigrams and bigrams which appear less than five times are eliminated. Other features or the combination of features can be considered input features, and there are no constraints on the features used by the proposed method. In this study, 2000 and 4000 reviews are selected randomly from IMDB and Amazon datasets, respectively.

### Evaluation measures

The proposed method is evaluated based on Precision, Recall, Accuracy, Specificity and F-measure^71^. Accuracy gives the percent of reviews whose attitudes were predicted correctly. Precision gives the percent of reviews estimated as positive attitudes correctly among the all reviews that are predicted as positive ones. Recall is the fraction of true estimated positive attitudes among all actual positive reviews and F-measure is the harmonic mean of precision and recall^72^. Specificity shows the percent of negative reviews that are predicted correctly.

## Results

For a fair comparison, tenfold cross-validation is applied to all experiments. The impact of SMSR is investigated by applying linear SVM on the above-mentioned features and comparing the obtained results with the following cases in numerous experiments:

1. Linear SVM with all input features, where regularization parameter, $\gamma$, was set by grid search between 11 equally logarithmically spaced points between ${10}^{-6}$ and ${10}^{-0.5}$.
2. Seven applied classifiers to sentiment analysis in the literature including $k$ NN, Naïve Bayesian (NB), Bagging, SVM with Radial Basis Function kernel (SVM(RBF)), Random Subspace, J48 and Random Forest under two situations. First, without applying any feature selection method, and second, by selecting suitable features using information gain, where the number of selected features is set to be {100, 200, 300, 400, 500, 600, 700, 800, 900, 1000}. So, eleven experiments have been done for each classifier. For more clarity, the best result of these experiments for each classifier is reported below. The classifier of WEKA (Waikato Environment for Knowledge Analysis, version 3.9.2, The university of Waikato, New Zealand) was exploited to apply above-mentioned classifiers^73^. The parameters were tuned according to Table 3.
3. It is worthwhile to mention that Random Subspace, J48 and Random Forest selects features in the nonlinear manner at the same time of training models. Therefore, they are suitable models for assessing the proposed method vs. nonlinear feature selection models.

**Table 3:** The parameter values of Classifiers.

Classifier	Parameter setting
$k$ NN	$k=1$
Random Forest	The number of trees = 100, the number of features to consider for training trees = log (the number of input features) + 1
Random Subspace	The size of each subspace = the half of all attributes,
Bagging	The size of each bag = the number of input data
J48	The minimum number of instances in each leaf = 2, The number of confidence threshold for pruning = 0.25
NB	–
SVM (RBF)	C = 1, $\gamma =1$

The SMSR’s parameters are set according to Table 4. The best values of $\lambda$ and $k$ for two datasets are obtained for 10 and 50, respectively. The best values of $d$ are 1500 and 2200 for IMDB and Amazon, respectively.

**Table 4:** The parameter values of SMSR.

Parameter	Range	Step size
$\lambda$	$[ {10}^{-2} {10}^{2}]$	10
$k$	$[10 200]$	10
$d$	[1000 2500]	100

**Figure 5:**
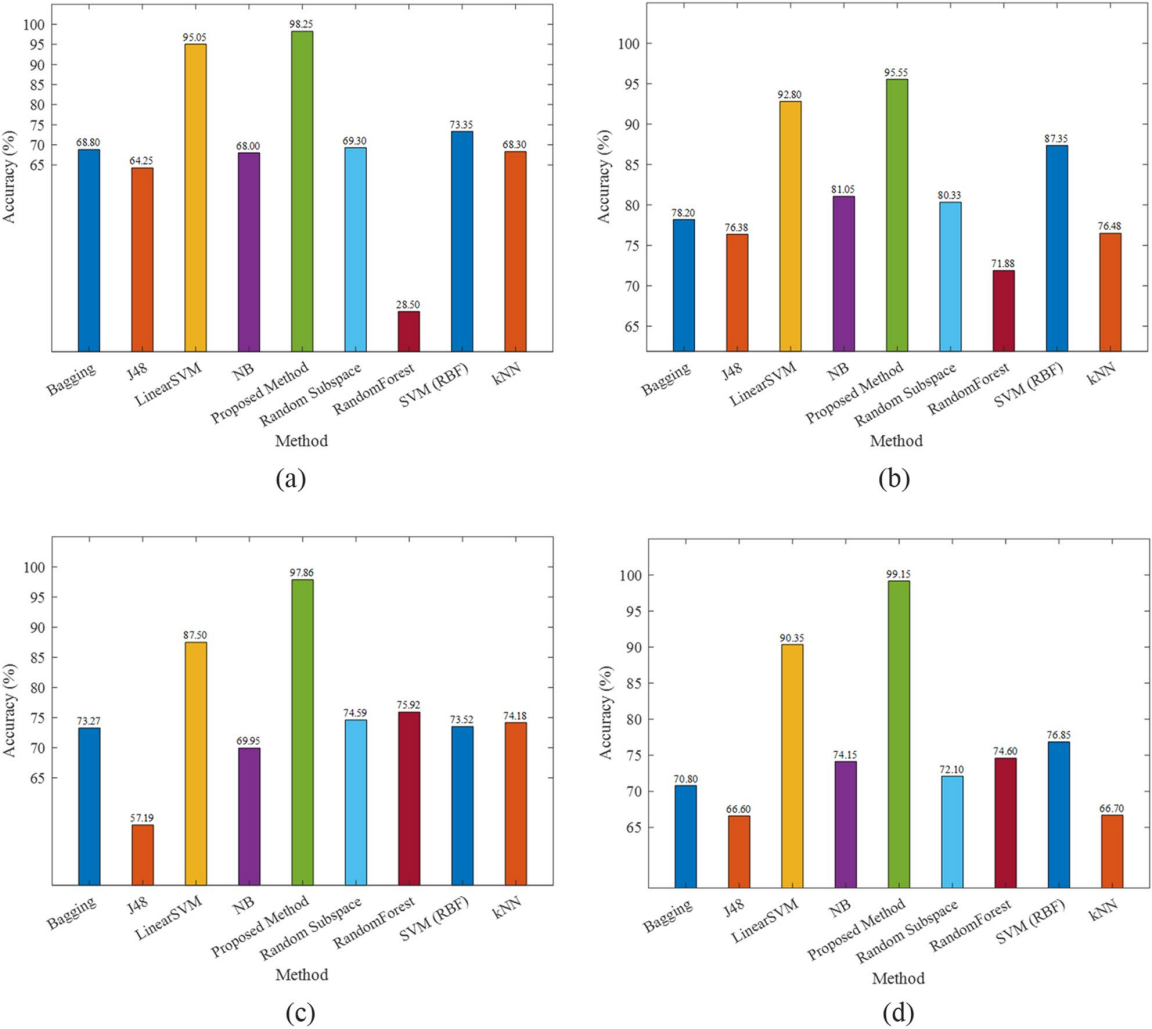
The accuracy of various methods on the IMDB dataset on (**a**) unigram + lexicon + tag features, (**b**) unigram features, (**c**) bigram features, and (**d**) unigram + lexicon features.

**Figure 6:**
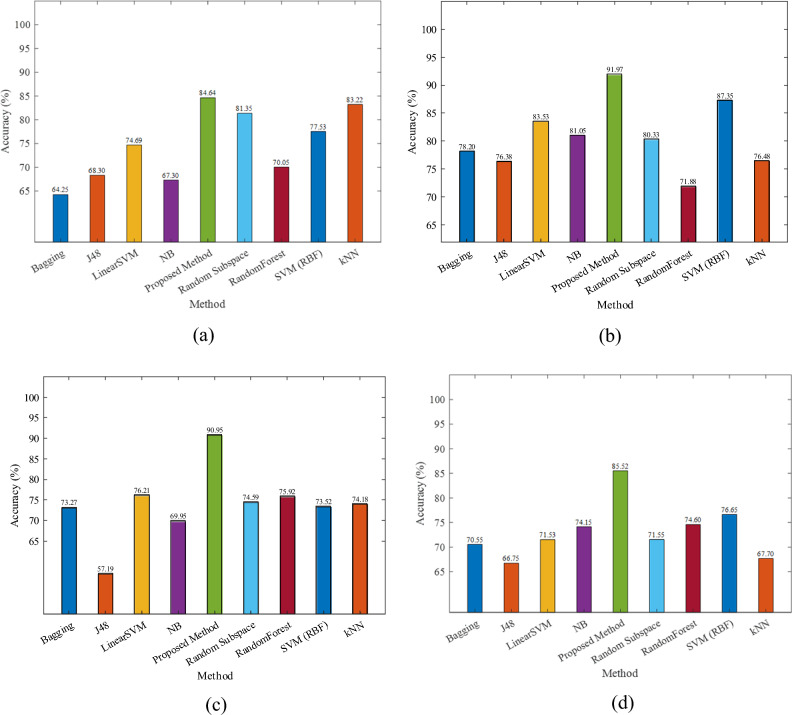
The accuracy of various methods on the Amazon dataset on (**a**) unigram + lexicon + tag features, (**b**) unigram features, (**c**) bigram features, and (**d**) unigram + Lexicon features.

The accuracy of forecasting the attitude of sentiments by various methods is summarized in Fig. 5 on IMDB dataset by considering unigram + lexicon + tag, unigram, bigram and unigram + lexicon. As shown in the figures, the accuracy of the proposed model with various input features is much higher compared to other methods. It showed that the new representation on unigram + lexicon input features has only 0.85% error on the IMDB dataset. The obtained performance measures on Amazon dataset are illustrated in Fig. 6 by considering unigram + lexicon + tag, unigram, bigram and unigram + lexicon, respectively. It showed that the lowest error, 8.03% is retained by unigram features on the Amazon dataset. The precision, recall, specificity and F1 of various methods on four input vectors are given in Table 5. The proposed method outperforms other methods in all performance measures. The best result on IMDB dataset is achieved exploiting both unigram + lexicon (F-Measure = 99.15, recall = 99.60) and bigrams (Precision = 99.68 and specificity = 99.69).

**Table 5:** The precision, recall, specificity and F1 of various methods for different input features on the IMDB dataset.

Original Features	Classifier Measure	Random Forest	J48	Random Subspace	SVM (RBF)	Bagging	NB	$k$ NN	Linear SVM	The proposed method
Unigram	Precision	85.40	78.60	83.00	89.20	80.30	81.50	83.50	93.64	**94.27**
	Recall	53.33	72.60	76.30	85.00	74.80	80.40	66.00	91.86	**97.00**
	Specificity	90.90	80.20	84.40	89.70	81.60	81.70	86.90	93.74	**94.09**
	F1	65.60	75.50	79.50	87.10	77.40	80.90	73.80	92.74	**95.62**
Unigram + lexicon + tag	Precision	74.30	65.60	71.30	81.00	71.70	72.60	73.90	94.83	**98.79**
	Recall	65.80	59.90	64.60	61.00	62.10	57.80	56.60	95.30	**97.70**
	Specificity	77.20	68.60	74.00	85.70	75.50	78.20	80.00	94.80	**98.80**
	F1	69.80	62.60	67.80	69.60	66.60	64.40	64.10	95.06	**98.24**
Bigram	Precision	70.10	54.20	68.60	66.90	67.40	64.60	67.60	90.37	**99.68**
	Recall	90.10	91.00	90.30	92.70	89.70	87.90	92.40	84.03	**96.03**
	Specificity	61.90	23.60	59.00	54.40	57.00	52.10	56.10	90.99	**99.69**
	F1	78.90	67.90	78.00	77.70	77.00	74.50	78.10	87.89	**97.82**
Unigram + lexicon	Precision	77.30	68.40	72.70	79.90	71.90	75.70	69.40	89.68	**98.71**
	Recall	69.70	61.70	70.70	71.80	68.20	71.10	59.80	91.20	**99.60**
	Specificity	79.50	71.50	73.50	81.90	73.40	77.20	73.60	89.50	**98.70**
	F1	73.30	64.90	71.70	75.60	70.00	73.30	64.20	90.43	**99.15**

Significance values are in bold.

Table 6 shows the precision, recall, specificity and F1 on Amazon dataset. The best result on the Amazon dataset is gained by unigram (F-measure = 92.16 and Recall = 94.12) and bigram (precision = 99.45 and specificity = 99.55). The obtained results are better than other methods in all performance measures on unigram + lexicon features. However, several methods have achieved the better results based on some measures, but the best F1 and accuracy are attained utilizing the proposed feature extraction method on all four type input features.

**Table 6:** The precision, recall, specificity and F1 of various methods for different input features on the Amazon dataset.

Original Features	Classifier	Random Forest	J48	Random Subspace	SVM (RBF)	Bagging	NB	$k$ NN	Linear SVM	The proposed method
Unigram	Precision	85.4	78.60	83.00	89.20	80.30	81.50	83.5	86.83	**90.28**
	Recall									
		53.33	72.60	76.30	85.00	74.80	80.40	66.00	79.18	**94.12**
	Specificity	**90.90**	80.20	84.40	89.70	81.60	81.70	86.90	87.91	89.80
	F1	65.60	75.50	79.50	87.10	77.40	80.90	73.80	82.83	**92.16**
Unigram + lexicon + tag	Precision	72.00	73.90	89.30	80.00	65.60	68.20	84.50	75.56	**82.17**
	Recall	65.70	56.60	71.20	73.40	59.90	64.70	81.50	72.96	**88.46**
	Specificity	74.40	80.00	**91.50**	81.60	68.60	69.90	85.00	76.42	80.82
	F1	68.70	64.10	79.30	76.60	62.60	66.40	82.90	72.24	**85.20**
Bigram	Precision	70.10	54.20	68.60	66.90	67.40	64.60	67.60	77.82	**99.45**
	Recall	90.10	91.00	90.30	**92.70**	89.70	87.90	92.40	72.63	82.17
	Specificity	61.90	23.60	59.00	54.40	57.00	52.10	56.10	79.72	**99.55**
	F1	78.90	67.90	78.00	77.70	77.00	74.50	78.10	87.89	**89.99**
Unigram + lexicon	Precision	77.30	68.80	72.80	79.90	71.30	75.70	72.80	74.59	**83.87**
	Recall	69.70	61.20	68.80	71.80	68.70	71.10	56.60	65.27	**87.95**
	Specificity	79.50	72.30	74.30	81.90	72.40	77.20	78.80	77.78	**83.10**
	F1	73.30	64.80	70.70	75.60	70.00	73.30	63.70	69.62	**85.86**

Significance values are in bold.

For more comparison, the proposed method is also compared with a manifold-based dimensionality reduction, where manifold is represented by sentiment and semantic analysis^23^. The graph is built by combining semantic and sentiment distance between reviews. The semantic distance metric is computed by a cosine similarity metric between opinion’s vectors, where Doc2Vec model^74^ has been utilized for representing opinions by vectors. The sentiment distance is computed by the valence aware dictionary for sentiment reasoning^75^. Isomap is applied for dimensionality reduction. Finally, Linear SVM with tenfold cross validation is applied to compare with the proposed method. The number of Doc2vec dimensions is selected from $\left\{300, 400, 500\right\},$ and the best results are reported in the Table 7. As the results show, the proposed method outperforms the method presented in^23^**.** However, the Doc2Vec model does not generate sparse vectors; the output vectors are high-dimensional yet. The proposed method can also discover the underlying structure of these data since it learns neighborhood from the data, as opposed to^23^ combines semantic and sentiment distance based on some fixed weights.

**Table 7:** The result of comparison of the proposed method with ^23^.

Dataset	Method	Precision	Recall	Specificity	F1	Accuracy
IMDB	^23^	78.30	64.60	82.10	70.79	73.35
	Proposed method	91.97	88.20	92.30	90.05	90.25
Amazon	^23^	64.94	56.35	69.59	60.34	62.97
	Proposed method	78.12	76.10	78.69	77.10	77.40

## Discussion

For more detailed analysis, the parameter sensitivity of the proposed method is assessed in this section. Three parameters may influence SMSR: $\lambda$, $k$, $d$. $\lambda$ indicates the importance of the sparsity of the solution in comparison to the reconstruction error, in which more values of $\lambda$ shows more sparsity of the solution. $d$ is the dimension of the output of SMSR and is limited to the number of data, since the number of eigenvalues of the Laplacian matrix is equal to data size. $k$ specifies the number of nearest data points in the input space to search for neighboring points in the output space.

**Figure 7:**
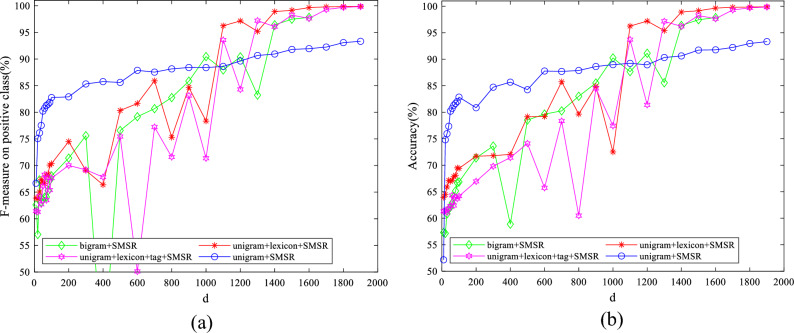
The impact of the parameter of $\mathrm{d}$ on (**a**) F-measure (**b**) accuracy of the proposed method on the IMDB dataset.

**Figure 8:**
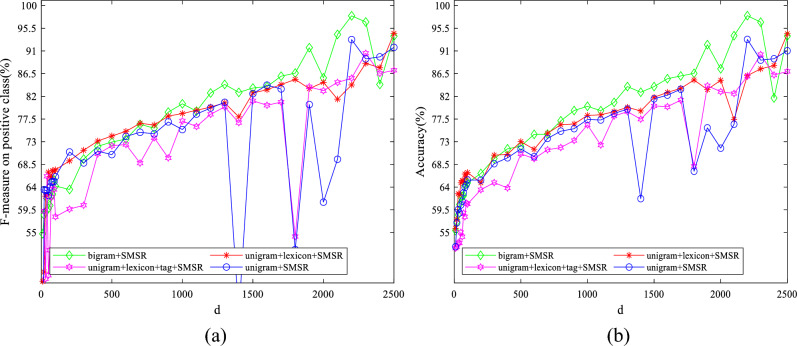
The impact of the parameter of $\mathrm{d}$ on (**a**) F-measure (**b**) accuracy of the proposed method on the Amazon dataset.

**Figure 9:**
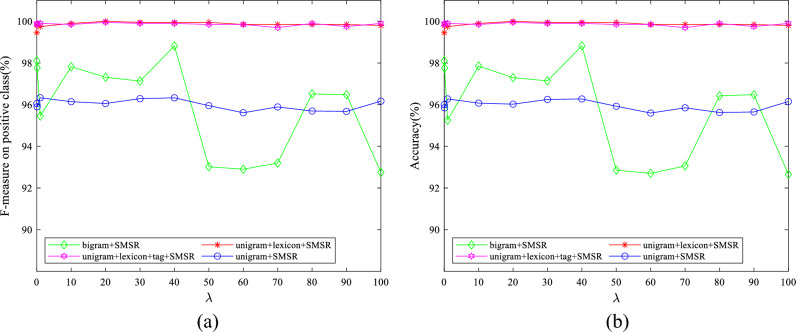
The impact of the parameter of $\uplambda$ on (**a**) F-measure (**b**) accuracy of the proposed method on the IMDB dataset.

**Figure 10:**
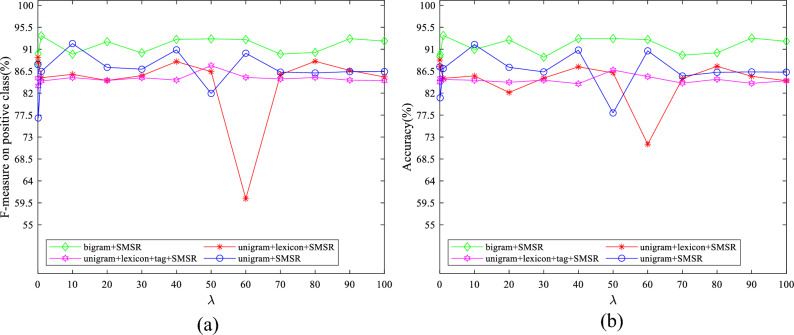
The impact of the parameter of $\uplambda$ on (**a**) F-measure and (**b**) accuracy of the proposed method on the Amazon dataset.

**Figure 11:**
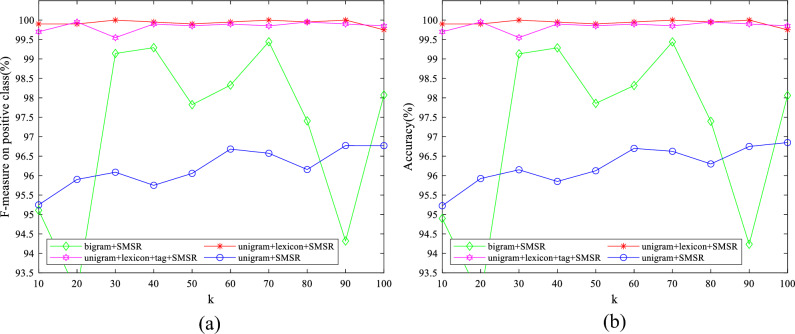
The impact of the parameter of $\mathrm{k}$ on (**a**) F-measure and (**b**) accuracy of the proposed method on the IMDB dataset.

To examine the effect of the value of parameters on the obtained results, some experiments have been conducted by varying them in a wide range for different features. The initial values of $\lambda$ and $k$ are set to 10 and 50, respectively. The initial value of $d$ is considered to be 1900 for IMDB dataset and 2500 for Amazon dataset. The initial variable settings are varied one by one to observe their effect on the performance. Figures 7–11 illustrate the impact of varying $d$, $\lambda$ and $k$ on the F-measure and accuracy measures on two considered datasets.

**Figure 12:**
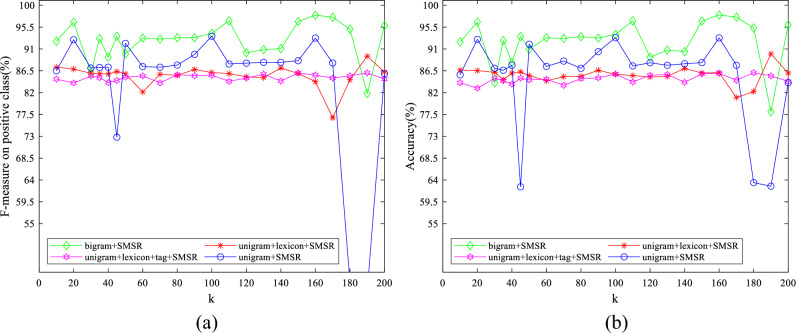
The impact of the parameter of $\mathrm{k}$ on (**a**) F-measure and (**b**) accuracy of the proposed method on the Amazon dataset.

Regarding Figs. 7, 8, it is observed that the models with large $d$ values achieve high performance. Consequently, linear SVM is exploited to classify data, which can deal with high dimensional data appropriately having fewer parameters than SVM with both polynomial and RBF kernels. As illustrated in Figs. 9, 10, $\lambda$ does not significantly affect the performance, which means that a sparse solution for each point from neighbors in the same manifold was successfully found for a wide range of values in the optimization problem. The standard deviation of accuracy in Fig. 9 is between 0.07% and 2.30%. As shown in Fig. 10, accuracy has a standard deviation of 0.75% and 1.71% for features learned from unigram + lexicon + tags and bigram, respectively. Figures 11, 12 illustrate the stability of the proposed method on varying $k$; although, both bigram and unigram + lexicon features may be affected by the values of this parameter compared to other features on IMDB dataset. Finally, the evaluated results reveal the strength of SMSR for different values of $k$ and $\lambda$ and a sufficiently large value of $d$. The effects of these parameters on other aforementioned performance measures are similar to F-measure and accuracy.

## Conclusion

The people’s opinion that are posted on social media platforms have an impact on a lot of users. Automated categorizing opinions into negative and positive feeling, utilizing machine learning and natural language processing techniques, is gaining significant importance. The short opinion texts convert into sparse high dimensional vectors, which lie on the intrinsic manifold structure. Discovering the manifold structure has a great effect on the accuracy of the obtained result. This paper investigated a sparse manifold representation of user-generated reviews by assuming lying data on some manifolds. The solution of the optimization problem, formulating these assumptions, concludes a graph of capturing the geometrical structure of data in a way that near data points from the same manifold connect with a higher weight than those from different manifolds. The spectral properties of the learned graph are exploited to portray a new representation of data.

The proposed representation method was applied to four features extracted from short unstructured opinion texts on two benchmark datasets. Many experiments have been conducted to explore the effectiveness of the manifold assumption and sparse property as prior knowledge in opinion mining in terms of accuracy, precision, recall, specification, and F-measure. The results revealed that the classification performance of the sentiments is ameliorated by utilizing the proposed feature extraction method. Specifically, the combination of manifold assumption and sparse property as prior knowledge in opinion mining leads to a better representation of opinions and a more accurate classification of sentiments. In addition, the impact of parameters is assessed in our experiments. The obtained results seem to be an excellent step toward better representation of opinions. Future studies can focus on enforcing the mentioned prior knowledge through deep learning-based seniment analysis.

## Data Availability

Data openly available in a public repository. IMDB and the subset used in this paper are available at http://www.cs.cornell.edu/people/pabo/movie-review-data/. The subset of Amazon review dataset is downloaded from https://drive.google.com/file/d/1EW-2ZiC2Df8PufsuNMPqIrdx6_zo29D1/view?usp=sharing (last access data: 9/21/2021).
